# Study of Vibronic and Cationic Features of m-Diethoxybenzene via REMPI, Hole-Burning, and MATI Spectroscopy

**DOI:** 10.3390/ijms262411818

**Published:** 2025-12-07

**Authors:** Xiateng Qin, Chunyang Duan, Yan Zhao, Changyong Li, Suotang Jia

**Affiliations:** 1 State Key Laboratory of Quantum Optics Technologies and Devices, Institute of Laser Spectroscopy, Shanxi University, Taiyuan 030006, China; 18734558738@163.com (X.Q.); 19003432874@163.com (C.D.); tjia@sxu.edu.cn (S.J.); 2 Department of Physics and Electronics Engineering, Jinzhong University, Jinzhong 030619, China; zhaoy@jzxy.edu.cn; 3 Collaborative Innovation Center of Extreme Optics, Shanxi University, Taiyuan 030006, China

**Keywords:** m-diethoxybenzene, rotamer, hole-burning, REMPI spectroscopy, MATI spectroscopy, adiabatic ionization energy, Franck–Condon spectral simulation

## Abstract

Phenetole derivatives are widely used in various fields, and the presence of the ethoxy group, with its longer alkyl chain, suggests the possibility of multiple rotamers. In particular, diethoxybenzene derivatives, containing two ethoxy groups, exhibit an even greater number of rotameric forms. In this study, we report the first investigation of the stable structures, vibronic spectra, and cationic spectra of different rotamers of m-diethoxybenzene (MDEB). Resonance-enhanced multiphoton ionization (REMPI) spectra of the rotamers were identified via hole-burning (HB) experiments, while mass-analyzed threshold ionization (MATI) spectra provided precise adiabatic ionization energies (IEs) of the observed rotamers, as well as the active vibrations of their corresponding cations. Density functional theory (DFT) calculations predicted thirteen rotamers of MDEB, but only two rotamers were observed in the supersonic molecular beam. The band origins of the S_1_ ← S_0_ transition and the adiabatic IEs of the down–up and down–down rotamers of MDEB were determined to be 36,091 ± 2 cm^−1^ and 36,165 ± 2 cm^−1^ and 62,419 ± 5 cm^−1^ and 63,378 ± 5 cm^−1^, respectively. Franck–Condon spectral simulations for the S_1_ ← S_0_ and D_0_ ← S_1_ transitions were performed based on DFT calculations, and the theoretical results showed good agreement with the experimental data. Vibrational features observed in the S_1_ and D_0_ states were assigned by comparing the experimentally measured spectra with the simulated spectra as well as the previously reported vibrational spectra of structurally similar molecules. Finally, several key findings and molecular characteristics are discussed in detail.

## 1. Introduction

Phenetole and its derivatives are widely applied in diverse fields, including medicine, materials science, agriculture, fragrances, liquid crystals, and electrochemistry. Due to the two torsional angles in its side chain, phenetole exists in multiple rotamers. Acting as “molecular fingerprints”, molecular spectra enable the accurate identification of molecules. High-resolution spectra of phenetole, covering its ground state, excited states, and ionic states, have been investigated using various techniques. Bernstein et al. first reported the resonance-enhanced multiphoton ionization (REMPI) spectra of phenetole [1], while Prampolini and colleagues conducted a density functional theory (DFT) study of the torsional potential in ethoxybenzene to run computer simulations of realistic models of liquid crystals containing alkoxy–aryl linkages, with four stable rotamers being identified [2]. Ferres and colleagues studied the molecular structure of phenetole via microwave spectroscopy and quantum chemical calculations, theoretically predicting two rotamers, trans and gauche; however, only the trans rotamer was observed in their supersonic jet experiment [3]. Egawa and colleagues measured the fluorescence excitation spectrum of the S_1_ ← S_0_ transition and performed theoretical calculations, and further suggested that both trans and gauche rotamers could be observed in their jet experiment of phenetole [4]. Chakraborty and colleagues measured the vibronically resolved fluorescence excitation and dispersed fluorescence spectra to demonstrate the existence of only one isomeric species of phenetole in the gas phase [5], and recently, Grotemeyer and colleagues studied the vibronic structure of the first electronically excited state S_1_ and ionic ground state D_0_ of trans phenetole via REMPI and mass-analyzed threshold ionization (MATI) spectroscopy [6]. Two signals, which are likely the origin bands of the two other rotamers, in a low-frequency direction of the 0^0^ band of the trans rotamer were observed in the REMPI spectrum.

For phenetole derivatives, more stable rotamers are expected. Tzeng and colleagues studied the excited state and cationic vibrational features of p-ethoxyphenol via REMPI and MATI spectroscopy and performed theoretical calculations to confirm that two rotamers of p-ethoxyphenol coexist in the sample [7]. Li et al. calculated the potential energy surface (PES) of 2-ethoxybenzonitrile in the ground state, and five stable rotamers were identified [8]. The most stable rotamer (trans) was observed in the supersonic molecular beam experiments, and the vibrational features of 2-ethoxybenzonitrile in the excited state and cationic ground state were measured by REMPI and MATI spectroscopy. m-Diethoxybenzene (MDEB) is an important phenetole derivative, but as far as we know, the spectra of its excited state and ionic ground state have not been reported in the literature.

MATI and zero-kinetic-energy (ZEKE) spectroscopy are currently the most popular high-resolution techniques for measuring vibrational features of cationic ground states. Tzeng et al. and Ketkov et al. used two-color MATI to study the cationic spectra of many benzene derivatives and sandwich molecules [9,10,11,12,13,14,15], while Kwon and colleagues built a vacuum ultraviolet single-photon MATI system to study many cationic vibrational features [16,17,18,19,20,21,22]. Yang et al. used two-color MATI, one-color MATI, and ZEKE technology to research cationic vibrational features of many metal clusters and molecules [23,24,25,26,27,28,29]. One-color MATI is suitable for probing the ionic vibrational features of species that contain only a single rotamer or exhibit extremely low transition probabilities for either the S_1_ ← S_0_ or D_0_ ← S_1_ transition. In contrast, two-color MATI spectroscopy enables the measurement of rotamer-specific ionic vibrational characteristics. Hole-burning spectroscopy stands out as one of the most efficient and crucial methods for rotamer discrimination, and it has been widely employed to resolve rotamers of numerous molecules or clusters [30,31,32,33,34,35,36,37]. In this work, we studied the vibronic and cationic features of m-diethoxybenzene via REMPI, hole-burning (HB), MATI spectroscopy, and theoretical calculations. Thirteen stable rotamers were identified theoretically, and hole-burning spectroscopy was used to resolve the vibronic features of specific rotamers in REMPI spectra. The measured spectra are assigned based on DFT calculations, simulated spectra, and assignments of structurally analogous molecules.

## 2. Results

### 2.1. Theoretical Rotamers of MDEB

**Figure 1:**
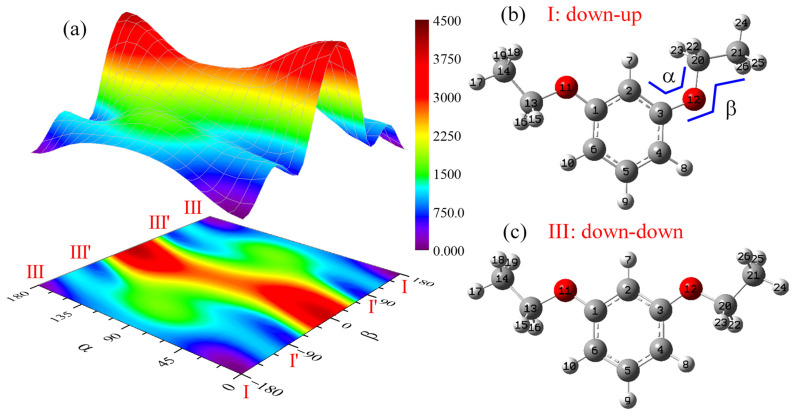
PES calculated at the B3LYP/6-311++g(d,p) level for the S_0_ state of MDEB (**a**) and its stable conformations (**b**,**c**).

The m-diethoxybenzene molecule contains two ethoxy groups. To explore its conformational landscape, we performed quantum chemical calculations at the B3LYP/6-311++g(d,p) level. One ethoxy group was fixed in the plane of the aromatic ring, while the two dihedral angles associated with the second ethoxy group were systematically scanned. This procedure yielded the two-dimensional potential energy surface (PES) of the electronic ground state shown in Figure 1a. The definitions of the two scanned dihedral angles, denoted as ∠α and ∠β, are illustrated in Figure 1b. The scan ranges for ∠α and ∠β were 0° to 180° and −180° to 180°, respectively.

The calculated PES reveals four distinct local energy minima. When ∠α is 0°, two minima (I and I’) are located, corresponding to ∠β values of approximately ±180° and ±90°, respectively. These minima represent configurations where the scanned ethoxy group is in-plane (∠β ≈ ±180°) or where its ethyl moiety is nearly perpendicular to the ring plane (∠β ≈ ±90°). Similarly, two additional minima (III and III’) are found when ∠α is 180°, also corresponding to ∠β ≈ ±180° and ±90°, respectively, indicating analogous in-plane and perpendicular ethyl orientations. However, these configurations (III and III’) are structurally distinct from I and I’. Thus, the PES confirms the existence of four stable rotamers.

The locations of these minima indicate that the molecular energy reaches a local minimum when ∠α is either 0° or 180° and when ∠β is +180° (which is equivalent to −180°), +90°, or −90°. In principle, this suggests six possible combinations of the dihedral angles for stable rotamers. However, the structures corresponding to ∠β = +90° and ∠β = −90° are mirror images of each other and are spectroscopically indistinguishable with the techniques employed in this study. Consequently, the statistical weight of these enantiomeric rotamers is doubled. For example, the statistical weights of rotamers I’ and III’ in Figure 1 are twice those of rotamers I and III.

In m-diethoxybenzene, each ethoxy group can adopt six distinct stable configurations based on combinations of its two dihedral angles. Consequently, with two such ethoxy groups, in principle, there are 6 × 6 = 36 possible combinations of the four dihedral angles that correspond to stable conformations. However, the spectroscopic techniques employed in this study cannot distinguish between enantiomeric structures, that is, mirror-image rotamers are considered identical in the spectral analysis. Nevertheless, their statistical weights are doubled, meaning that the observed spectral intensities for such pairs will be twice what would be expected if statistical weights were not accounted for.

Due to the large number of rotamers in m-diethoxybenzene, clear differentiation and systematic naming are essential. Our nomenclature follows the established convention for m-dimethoxybenzene, m-methoxyphenol, and resorcinol, as reported in [38,39,40]. These studies designate the three rotamers as rotamer I (down–up), rotamer II (up–up), and rotamer III (down–down), where “up” and “down” refer to the relative orientation of the methoxy or hydroxyl group. A similar naming scheme is applied in Figure 1b,c of the present work.

**Figure 2:**
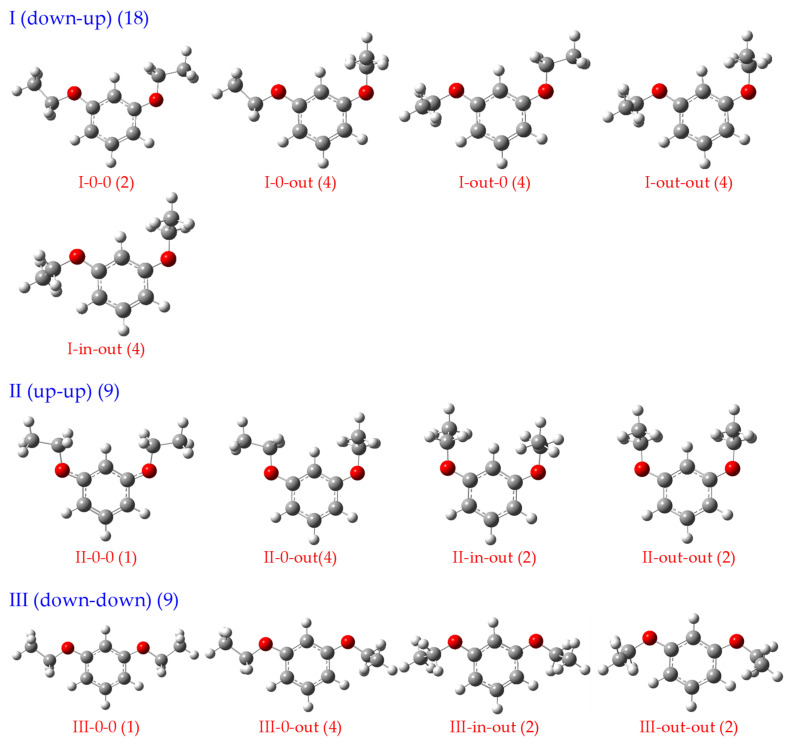
All 13 possible stable rotamers and their respective statistical weights (given in parentheses).

For m-diethoxybenzene, variations in the two dihedral angles ∠α yield three fundamental structural types analogous to those in m-dimethoxybenzene: I (down–up), II (up–up), and III (down–down). The full set of stable conformations is then generated by the various combinations of the two dihedral angles ∠β. The ethyl group in each ethoxy moiety can assume three allowed orientations: in the plane of the ring (denoted as “0”), outward from the plane of the paper (“out”), or inward toward the plane of the paper (“in”). All 13 resulting stable rotamers, along with their respective statistical weights (given in parentheses), are presented in Figure 2, with the sum of the weights being 36.

To compare the relative proportions of each rotational rotamer in the supersonic molecular beam, it is necessary to determine the electronic ground-state energies of each rotamer. We computed these energies using four different methods: B3LYP/6-311++g(d,p), B3PW91/6-311++g(d,p), B3LYP/aug-cc-pvtz, and B3PW91/aug-cc-pvtz. The results, summarized in Table 1, show that the down–up–0–0 (i.e., I–0–0), down–down–0–0 (i.e., III–0–0), and up–up–0–0 (i.e., II–0–0) rotamers exhibit the lowest energies, which is consistent with the behavior of m-dimethoxybenzene. In these three rotamers, both ethoxy substituents lie in the plane of the aromatic ring. For simplicity, these are referred to in the following sections as down–up (I), down–down (III), and up–up (II). Owing to lowest energy and a statistical weight of 2 of rotamer I, compared to a weight of 1 for both rotamers III and II, it is expected to have the highest population according to the Maxwell–Boltzmann distribution. As a result, the spectral signal of rotamer I is generally the most intense.

### 2.2. Vibronic and Hole-Burning Spectra of MDEB

**Figure 3:**
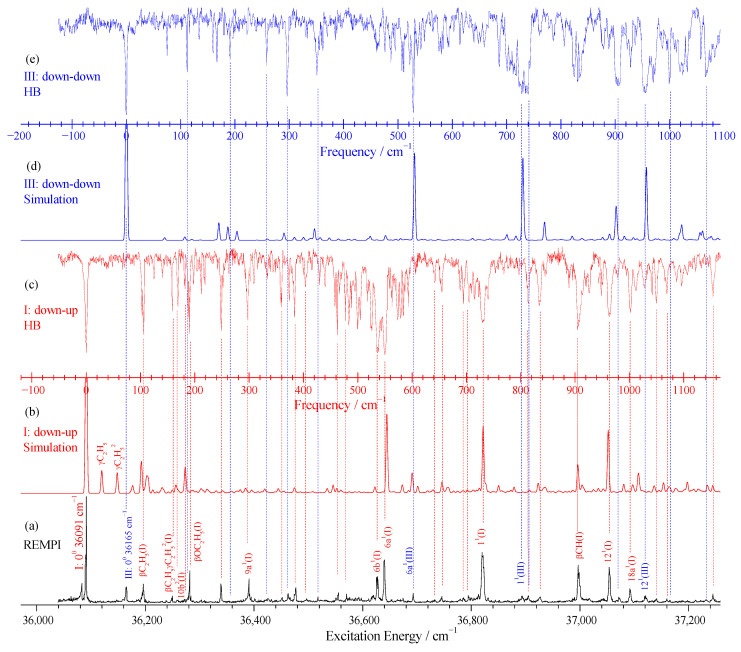
The two-color R2PI spectrum of MDEB is shown in (**a**). The HB spectra are presented in (**c**,**e**), where the probe lasers are fixed at the transition origins of 36,091 and 36,165 cm^−1^, respectively. The simulation spectra of the S_1_ ← S_0_0^0^ transition for the down–up and down–down rotamers are shown in (**b**,**d**), respectively.

The two-color resonance-enhanced two-photon ionization (R2PI) spectrum of m-diethoxybenzene is presented in Figure 3a. Because theoretical calculations indicate the existence of multiple rotational rotamers, the observed R2PI spectrum therefore represents a superposition of contributions from several rotamers. To resolve the spectral features of individual rotamers, hole-burning (HB) spectroscopy was employed [41]. When the strongest peak at 36,091 cm^−1^ was used as the probe signal and the hole-burning laser wavelength was scanned, the hole-burning spectrum shown in Figure 3c was obtained. Clearly, most of the vibrational features observed in the REMPI spectrum exhibit corresponding holes in the hole-burning spectrum. For the REMPI peaks without matching hole-burning signals, notably the relatively strong peak at 36,165 cm^−1^, this signal was selected as a new probe to scan the hole-burning laser, yielding the spectrum in Figure 3e. Except for the peak at 36,083 cm^−1^, other REMPI peaks have a one-to-one correspondence in the hole-burning spectra shown in Figure 3c,e, indicating the presence of two detectable rotamers of m-diethoxybenzene under the supersonic jet conditions of this study. Based on this, the stronger REMPI signal with an origin at 36,091 cm^−1^ is assigned to the down–up (I) rotamer, which has the lowest ground-state energy, while the REMPI signal originating at 36,165 cm^−1^ is attributed to the down–down (III) rotamer, which has the second lowest ground-state energy.

As for the peak at 36,083 cm^−1^, it should be a hot band. A similar band has also been observed in the REMPI spectrum of 1,3-dimethoxybenzene [42,43]. It likely does not originate from the up-up rotamer, although the theoretically calculated energy of the up-up rotamer is approximately 220 cm^−1^ higher than that of the down-up rotamer. However, the statistical weight of the down-up rotamer is twice that of the up-up rotamer, resulting in a smaller population of molecules in the up-up rotamer, which makes it difficult to detect. Moreover, in structurally similar molecules such as resorcinol [44], 3-methoxyphenol [39], and 1,3-dimethoxybenzene [43,45], the energy of the up-up rotamer is also about 200 cm^−1^ higher than that of the lowest-energy rotamer. Nevertheless, no recent literature has reported the discovery of the up-up rotamer; instead, it has been confirmed that the previously proposed up-up bands actually belong to the down-up rotamer [45].

To assign the measured vibrationally resolved spectra of the excited states for each rotamer, we simulated the S_1_ ← S_0_ transition spectra. For comparison, the simulated spectra of the down–up and down–down rotamers are displayed in Figure 3b and Figure 3d, respectively. Good agreement is observed between the experimental hole-burning spectra and the simulated spectra. Based on the simulations and by analogy with structurally related molecules, such as m-dimethoxybenzene [43], m-methoxyphenol [39], resorcinol [44,46], and vibrational bands observed in the REMPI experiment were assigned. The excitation energies, measured and computed vibrational frequencies, and mode assignments for both rotamers are summarized in Table 2. The numbering system for the normal vibrations follows that used by Varsányi and Szoke [47,48] for benzene derivatives, which is based on Wilson’s notations [49].

For the down–up rotamer, strong vibrational bands appear at 549, 729, and 963 cm^−1^, which are assigned to the fundamental modes 6a, 1, and 12, respectively. These all involve in-plane distortions of the carbon ring, which is consistent with ring expansion upon electronic excitation. Several other in-plane vibrational modes and combination bands of the aromatic ring were also observed. For example, bands at 300, 386, 465, 534, 1001, and 1154 cm^−1^ are assigned to modes 9a, 15, 11, 6b, 18a, and the combination band 6a^1^19a^2^, respectively. In-plane rocking motions of the substituents were identified as well: bands at 105, 191, and 906 cm^−1^ are attributed to β(C_2_H_5_), β(OC_2_H_5_), and β(CH) of the methyl group, respectively.

For the down–down rotamer, the REMPI signals are relatively weak. Nevertheless, both the hole-burning and simulated spectra indicate that, similar to the down–up rotamer, the fundamental modes 6a, 1, and 12 are strongly active, appearing at 528, 730, and 954 cm^−1^, respectively. The in-plane ring mode 9a is observed at 298 cm^−1^, while the in-plane rocking vibration of the ethyl group, β(C_2_H_5_), appears at 117 cm^−1^, and the in-plane methyl C–H bending vibration, β(CH) (at CH_3_), is observed at 907 cm^−1^.

### 2.3. Cationic Spectra of MDEB

To the best of our knowledge, information concerning the cationic vibrations of m-diethoxybenzene is not available in the literature. Consequently, we conducted MATI experiments to determine the adiabatic IEs and cationic vibrational frequencies of selected rotamers of this molecule with high precision.

**Figure 4:**
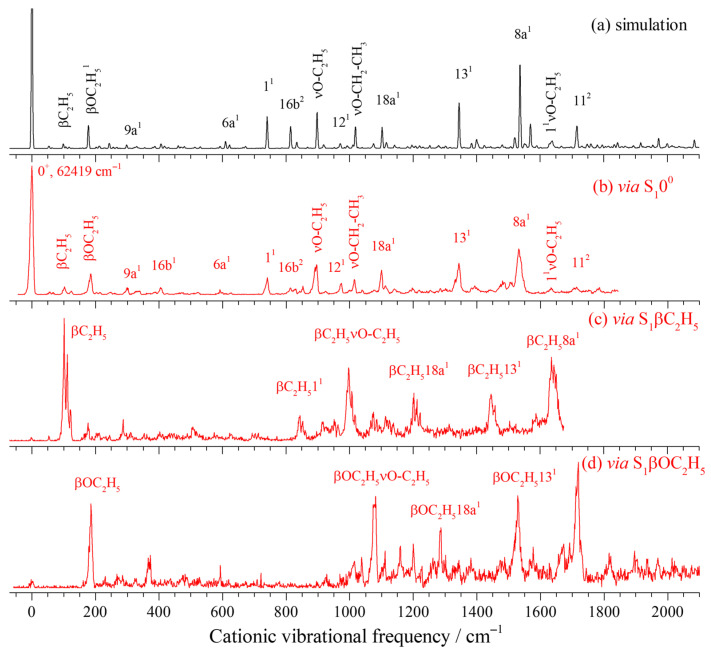
Simulated spectrum of the D_0_ ← S_1_0^0^ transition of the MDEB down–up rotamer (**a**) and its MATI spectra via the intermediate states S_1_0^0^ (**b**), S_1_βC_2_H_5_ (**c**), and S_1_βOC_2_H_5_ (**d**).

Figure 4a–d present the simulated spectrum of the D_0_ ← S_1_0^0^ transition for the down–up rotamer, along with the MATI spectra obtained via the S_1_0^0^, S_1_βC_2_H_5_, and S_1_βOC_2_H_5_ intermediate states. The simulated spectrum shows good agreement with the MATI spectrum via the S_1_0^0^ state. Based on the simulated spectrum and spectral assignments of structurally analogous molecules (such as m-dimethoxybenzene [43], m-methoxyphenol [39], and resorcinol [44,46]), we assigned the electronic–vibrational spectrum of the cation of the down–up rotamer measured in the experiment. Several in-plane vibrations of the carbon ring were observed, such as strong cationic vibrations appearing at 741, 974, 1100, 1344, and 1531 cm^−1^, which were assigned to the fundamental modes 1, 12, 18a, 13, and 8a, respectively. These all correspond to in-plane distortion motions of the carbon ring, which are associated with ring contraction induced by the excitation process. Weaker in-plane ring vibrations 9a, 6b, and 6a were also observed at 299, 520, and 591 cm^−1^, respectively. A small number of out-of-plane vibrational modes were detected with relatively weak signals. For example, the fundamental out-of-plane vibration 16b^1^, its second overtone (16b^2^), and other out-of-plane modes 11^1^ and 11^2^ were identified at 405, 814, 852, and 1713 cm^−1^, respectively. The in-plane rocking and stretching vibrations (βC_2_H_5_, βOC_2_H_5_, νO–C_2_H_5_, and νO–CH_2_–CH_3_) of the substituent were observed at 102, 186, 895, and 1014 cm^−1^, respectively. A combination band involving ring breathing and substituent stretching, labeled 1^1^νO–C_2_H_5_, was identified at 1633 cm^−1^.

Figure 4c,d display the MATI spectra obtained via the S_1_βC_2_H_5_ and S_1_βOC_2_H_5_ intermediate states, with thorough comparisons revealing that these spectra exhibit profiles similar to those in Figure 4b. Further analysis indicates that the vibrational bands observed in these two MATI spectra can be interpreted as combination bands involving vibrational modes of the respective intermediate states and those observed in Figure 4b, with no new fundamental mode being activated.

**Figure 5:**
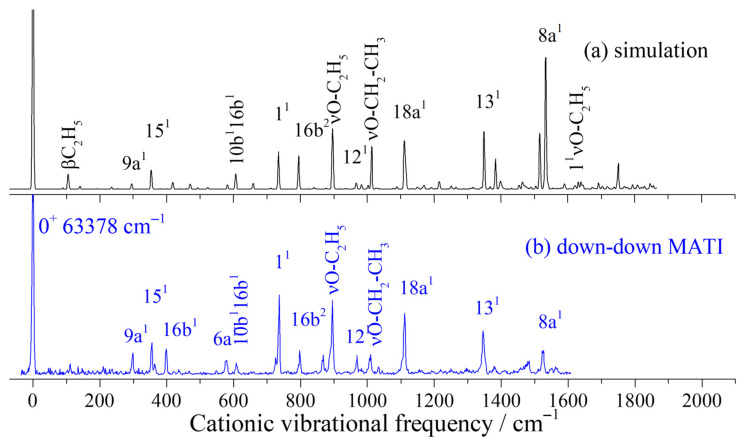
Simulated spectrum of the D_0_ ← S_1_0^0^ transition of the MDEB down–down rotamer (**a**) and its MATI spectra via the intermediate state S_1_0^0^ (**b**).

Figure 5a,b show the simulated spectrum of the D_0_ ← S_1_0^0^ transition and the MATI spectrum via the S_1_0^0^ intermediate state for the down–down rotamer. Apart from the weaker intensity in the high-frequency region of the experimental MATI spectrum compared to the simulation, good agreement is obtained between theoretical and experimental results. The weaker signal in the high-frequency region of the MATI spectrum is attributed to the decreasing energy of the dye laser toward shorter wavelengths; the data were not corrected for variations in laser energy across the wavelength range. Thus, the attenuation at higher frequencies arises from experimental limitations. Therefore, the theoretical simulation serves as an equally valuable reference for assigning the MATI spectrum of the down–down rotamer, as in the case of the down–up rotamer. In-plane distortion vibrational modes (9a, 15, 6a, 1, 12, 18a, 13, and 8a) of the carbon ring were observed at 298, 356, 579, 736, 969, 1110, 1346, and 1523 cm^−1^, respectively. The out-of-plane vibrational mode 16b and its overtone were also activated, with frequencies of 399 and 797 cm^−1^. Strong ethoxy stretching vibrations, νO–C_2_H_5_ and νO–CH_2_–CH_3_, were detected at 895 and 1009 cm^−1^, respectively, and other weak vibrations and combination bands that were observed are listed in Table 3.

## 3. Discussion

### 3.1. Effect of Substituent Orientation on Energy

The simulated spectrum of m-diethoxybenzene demonstrates good consistency between theoretical calculations and experimental results, indicating that the theoretical findings hold significant reference value for investigating molecular structure, energy, and related aspects. Based on the theoretically calculated energies of each rotamer in Table 1, the regularities below are found.

The electronic ground-state energies of molecular rotamers calculated by four different methods show slight differences, while the order of energy magnitudes remains strictly consistent.

Regardless of whether both ethoxy groups lie within the ring plane, one lies within the ring plane and the other is out of the plane, or both are out of the plane, the ground-state energies of rotamers in each case adhere to the following order: down–up < down–down < up–up.

The configuration energy when the C-C bond of a single ethoxy group is out of the ring plane is approximately 600 cm^−1^ higher than when the C-C bond lies within the ring plane. If the C-C bonds of both ethoxy groups are out of the ring plane simultaneously, the energy is approximately 1200 cm^−1^ higher than when both C-C bonds lie within the ring plane.

When the C-C bonds of the two substituents are both out of the ring plane, the configuration energy when they are on the same side of the ring plane is slightly higher than that when they are on opposite sides. For the down–up and down–down configurations, this energy difference is approximately 0–3 cm^−1^, while for the up–up configuration, the ethoxy groups are relatively close to each other, potentially leading to intramolecular interactions. Consequently, the energy of the same-side configuration is approximately 10–30 cm^−1^ higher than that of the opposite-side configuration.

### 3.2. Molecular Geometry in the S_0_, S_1_, and D_0_ States

Table 4 and Table 5 list the geometric parameters of the down–up and down–down rotamers of MDEB in the S_0_, S_1_, and D_0_ states, which were calculated at the B3PW91/aug-cc-pvtz, TD-B3PW91/aug-cc-pvtz, and UB3PW91/aug-cc-pvtz levels, respectively. They also show that each C-C bond of the ring becomes longer following the S_1_ ← S_0_ transition, with the ring circumference (sum of the six bond lengths) increasing by 0.145 Å and 0.150 Å. In contrast, during the D_0_ ← S_1_ transition, the C-C bond lengths of the ring do not change consistently: some increase (C3-C4 and C6-C1) while others shorten, resulting in ring circumference changes of −0.071 Å and −0.068 Å, respectively (note: a negative value indicates shortening). For all C-H bonds (which are not listed in the tables for simplicity), the bond length does not significantly change. These trends are consistent with those of other benzene derivative molecules [50,51,52]. The variation in the bond angles is limited to the −4.28° to 2.74° range, whereas the dihedral angles of the substituents for the down–up rotamer only vary between −1.89° and 1.89°. The down–down rotamer possesses C2v symmetry, and thus, its dihedral angles remain unchanged. These results indicate high transition probabilities between the ground state S_0_, the excited state S_1_, and the ionic ground state D_0_, which have been attributed to the similar molecular structures of these states. This is supported by strong REMPI and MATI signals. The structural variation in the aromatic ring during the transitions leads to the activation of numerous in-plane vibrational modes.

### 3.3. Effect of Substituent Orientation on Vibrational Frequencies

**Figure 6:**
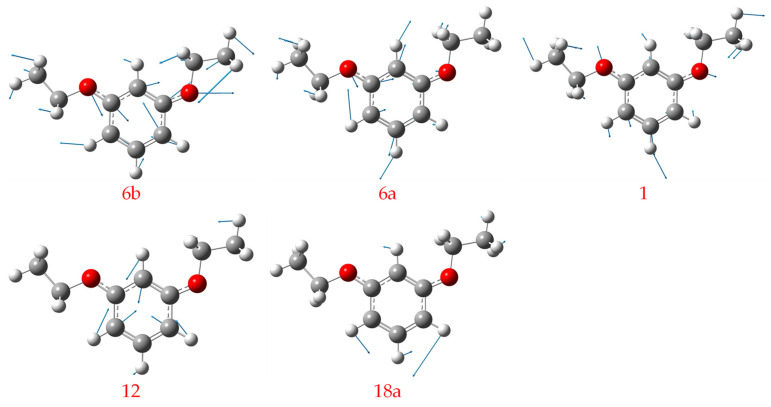
The patterns of the five typical vibrational modes of the MDEB down–up rotamer D_0_ state.

The several vibrational modes of the down–up rotamer identified in the experiment are illustrated in Figure 6. These vibrational patterns are typical and easy to assign or identify. To compare the vibrational frequency differences between m-diethoxybenzene and m-dimethoxybenzene, the fundamental frequencies of five vibrational modes (6b, 6a, 1, 12, and 18a) for the down–up and down–down rotamers of these two molecules are listed in Table 6. The theoretically calculated values are in parentheses, and the measured values of m-dimethoxybenzene can be found in [43].

As observed in Table 6, except for the theoretically calculated value of the S_1_18a mode in the down–down configuration, the vibrational frequencies of m-diethoxybenzene are consistently greater than or equal to those of m-dimethoxybenzene for the corresponding vibrational modes of the same rotamer in either the excited state S_1_ or the ionic ground state D_0_. According to the oscillator frequency formula, ν = 1/(2π)√(k/μ), where k is the force constant of the potential energy surface at the equilibrium internuclear distance and μ is the reduced mass. For most vibrational modes, the reduced mass μ of m-diethoxybenzene is larger than that of m-dimethoxybenzene. However, the ethoxy group OC_2_H_5_ exhibits higher spatial rigidity and a stronger electron-donating ability than the methoxy group OCH_3_. This enhances the bonding interaction of the benzene ring skeleton and increases the vibrational constraint, resulting in a larger force constant k and thus a higher vibrational frequency.

From the data in Table 6, the theoretically calculated frequencies are in good agreement with the experimentally measured ones. Although some vibrational modes were not observed in the experiment, the theoretical values should still effectively reflect the actual situation. Except for the vibrational frequency of the 6b mode in the ionic state of the down–down rotamer, which is slightly lower than that in the S_1_ state, the vibrational frequencies of the ionic states of all other rotamers are higher than those of the excited states. In particular, the vibrational frequency of the 18a mode in the cation is approximately 100 cm^−1^ higher than that in the excited state, while the theoretically calculated vibrational frequency of the 6b mode in the cation of the down–down rotamer of m-diethoxybenzene is 115 cm^−1^ higher than that in the excited state, indicating that the cation has higher rigidity than the excited state.

For the two different configurations of the same molecule, the vibrational frequencies of modes 6a, 1, 12, and 18a are relatively close. However, significant differences are observed for the 6b mode: the theoretically calculated vibrational frequencies of the 6b mode in the excited state S_1_ and ionic ground state D_0_ of the down–up configuration of m-dimethoxybenzene are approximately 96 cm^−1^ and 85 cm^−1^ higher than those of the down–down configuration, respectively. For m-diethoxybenzene, the theoretically calculated vibrational frequency of the 6b mode in the excited state of the down–up configuration is 88 cm^−1^ higher than that of the down–down configuration. This indicates that different orientations of the substituents cause substantial changes in the vibrational frequency of the 6b mode.

### 3.4. Transition Energy and Ionization Energy

Transition energy (TE) and ionization energy (IE) are important characteristic parameters of molecules, and the theoretical calculation of these energies provides a significant reference value for distinguishing rotamers. TD-BPW91/aug-cc-pvtz calculations indicate that the excitation energy of the down–up rotamer is 4.6936 eV (i.e., 37,856 cm^−1^), and that of the down–down rotamer is 4.7318 eV (i.e., 38,165 cm^−1^), indicating that the calculated transition energy (TE) of the down–down rotamer is 309 cm^−1^ higher than that of the down–up rotamer, which is consistent with the assignment of the down–up and down–down rotamers in Section 2. The IEs calculated using density functional theory (DFT) exhibit large errors and therefore have limited reference value for related research works. CBS-QB3 and G4 methods generally yield relatively accurate IEs, and these two methods were employed to calculate the IEs of the rotamers: the IEs of the down–up and down–down rotamers calculated using CBS-QB3 are 62,972 cm^−1^ and 64,006 cm^−1^, respectively, while those calculated using the G4 method are 62,008 cm^−1^ and 63,004 cm^−1^, respectively. Both methods predict that the IE of the down–up rotamer is approximately 1000 cm^−1^ lower than that of the down–down rotamer, which is in good agreement with the experimentally measured IEs of 62,419 cm^−1^ (down–up) and 63,378 cm^−1^ (down–down). The CBS-QB3 calculations overestimate the experimental values, with relative errors of +0.89% and +0.99%, respectively; in contrast, the G4 calculations underestimate the experimental values, with relative errors of −0.66% and −0.59%, respectively. Although the G4 method provides more accurate results, it consumes more computational resources. The theoretically calculated IEs are in good agreement with the experimentally measured ones.

The experimentally measured IEs of the down–up and down–down configurations of m-diethoxybenzene are 62,419 cm^−1^ and 63,378 cm^−1^, respectively, which are 1104 cm^−1^ and 1113 cm^−1^ lower than the IEs of the corresponding configurations of m-dimethoxybenzene (63,523 cm^−1^ and 64,491 cm^−1^ [43], respectively). This indicates that the long-chain effect (i.e., the longer carbon chain of the ethoxy group compared to the methoxy group) leads to a reduction in ionization energy.

## 4. Experimental and Theoretical Methods

### 4.1. Experimental Methods

The liquid sample of 1,3-diethoxybenzene (purity 97%), purchased from Shanghai Xian Ding Biotechnology Co., Ltd., (Shanghai, China), and used without further purification, was heated to ~80 °C to achieve sufficient vapor pressure. Krypton (Kr) is used as a carrier gas at a backing pressure of 3 bar. The gas mixture, seeded with sample molecules, expanded into the source chamber through a pulsed valve (Parker Series 9 General Valve, Parker Hannifin Corporation, Cleveland, OH, US) with a nozzle orifice diameter of 0.8 mm. The operating pressures in the source and ionization chambers were approximately 1.1 × 10^−3^ Pa and 1.2 × 10^−5^ Pa, respectively. The supersonic molecular beam was collimated by a 1 mm diameter skimmer before entering the ionization chamber, where it interacted with laser radiation to prepare molecules in the desired excited and ionic states. The resulting ions traveled a free-flight distance of approximately 48 cm before being detected by a pair of stacked microchannel plates (MCPs; 25 mm diameter, Shanxi Great Wall Microelectronics Co., Ltd., Taiyuan, China). The ion signals were subsequently collected and processed.

For the REMPI experiments, two laser systems were employed: The first system consisted of a PrecisionScan-D dye laser (using Coumarin 153, Exciton Inc., Dayton, OH, USA. All dyes used in this study are sourced from this company) pumped by the third harmonic (355 nm) of a Q-smart 850 Nd:YAG laser (Quantel, Les Ulis, Essonne, France). The second system comprised a CBR-D-24 dye laser (using Pyrromethene 580) pumped by the 532 nm output of an INDI-40-10 Nd:YAG laser (Newport Corp., Irvine, CA, USA). The outputs of the two dye lasers (which were operated simultaneously) were further frequency-doubled using BBO crystals to generate ultraviolet lasers. The relative timing of the pulse valve, YAG laser, pulse voltage, and signal acquisition system was precisely controlled by a digital delay generator (DG645, Stanford Research Systems, Inc., Sunnyvale, CA, USA) with eight delayed output channels. To acquire the REMPI spectrum, the wavelength of the CBR-D-24 dye laser (ionization laser) (Sirah Lasertechnik GmbH, Göttingen, Lower Saxony, Germany) system was fixed at 285 nm to ionize excited-state molecules, while the output wavelength of the PrecisionScan-D dye laser (excitation laser) (Sirah GmbH, Göttingen, Germany) was scanned. The REMPI measurements over a ~1200 cm^−1^ range were performed via segmented scanning, with each segment covering 5–10 nm. Neutral density filters were used to adjust the laser intensity, ensuring that the energies of the excitation laser and the ionization laser remained approximately 10 μJ and 100 μJ, respectively, throughout the entire scanning range.

UV-UV hole-burning spectroscopy was employed to determine the number of rotational rotamers (rotamers) present in the supersonic beam. This technique uses a strong “pump” (or “burn”) laser (~1.5 mJ) and a weaker “probe” laser (~10 μJ), which sequentially interact with the molecular sample with a controlled delay of approximately 150 ns. To obtain a hole-burning spectrum, the probe laser wavelength was fixed on a specific peak (e.g., the electronic origin) of the REMPI spectrum, while the pump laser wavelength was scanned. When the pump laser was resonant with a vibronic transition of a specific rotamer (e.g., rotamer I), the intense pump radiation depleted the ground-state population of that rotamer via excitation and subsequent ionization, causing a corresponding decrease in the ion signal generated by the probe laser. A minimum in the probe ion signal thus indicated a vibrational level of the targeted rotamer, building up its specific hole-burning spectrum. By repeating this procedure with the probe laser fixed on different REMPI peaks, the hole-burning spectra of other rotamers could be recorded. The hole-burning spectra exhibited broader linewidths due to the high laser fluence used. Comparisons between the REMPI and hole-burning spectra allowed for the assignment of spectral features to individual rotamers and the determination of the number of rotamers present in the molecular beam.

For the two-color MATI experiments, one laser system consisted of a PrecisionScan-D dye laser (using LDS 722 or LDS 751 dye in ethanol) pumped by the 532 nm output of a Q-smart Nd:YAG laser. The other system was the CBR-D-24 dye laser (Pyrromethene 580) pumped by the 532 nm output of the INDI-40-10 Nd:YAG laser.

In this scheme, molecules were excited by two tunable UV lasers to long-lived high-n Rydberg states. Approximately 100 ns after the laser pulses, a pulsed electric field of –0.7 V/cm was applied to deflect directly formed ions, but the high-n Rydberg molecules remained largely unaffected and continued their flight. After a delay of ~32 μs, which allowed the Rydberg molecules to enter a designated field-ionization region, a strong pulsed electric field of +143 V/cm was applied for 50 μs. This field ionized the Rydberg states via the Stark effect. The resulting ions were mass-analyzed, and the signals were processed using a multichannel scaler (Stanford Research Systems SR430, Stanford Research Systems, Inc., Sunnyvale, CA, USA) before being transferred to a computer. Data points were averaged over 300 laser shots per wavelength, and the absolute wavelengths of both dye lasers were calibrated using a wavemeter (HighFinesse WS-7, HighFinesse GmbH, Offenburg, Germany). Further details on the experimental system have been described in our previous publications [8,41,53,54].

The measurement errors of the vibrational frequencies and IEs are consistent with those obtained under typical experimental conditions, i.e., ±2 cm^−1^ and ±5 cm^−1^, respectively.

### 4.2. Theoretical Methods

All calculations were performed using the Gaussian 16 program package [55]. Geometry optimizations and vibrational frequency calculations for the ground (S_0_), first electronically excited (S_1_), and cationic ground (D_0_) states were conducted at the B3PW91/aug-cc-pvtz, TD-B3PW91/aug-cc-pvtz, and UB3PW91/aug-cc-pvtz levels of theory, respectively. The molecular ground-state potential energy surface was calculated at the B3LYP/6-311++g(d,p) level, and the electronic ground-state energies of the 13 identified rotamers were computed using four different methods: B3LYP/6-311++g(d,p), B3PW91/6-311++g(d,p), B3LYP/aug-cc-pvtz, and B3PW91/aug-cc-pvtz. Prior to the experiments, the Gaussian 16 program was also used to predict ionization energies via the G4 and CBS-QB3 composite methods, providing essential data for the selection of appropriate laser dyes. Spectral simulations were carried out based on the B3PW91/aug-cc-pvtz computational results.

## 5. Conclusions

The theoretical calculations of the ground-state potential energy surface for m-diethoxybenzene were performed, and a detailed analysis of the stable configurations formed by different orientations of the two ethoxy groups identified 13 stable rotamers. The energies and statistical weights of these configurations were calculated and compared, finding that the electronic ground-state energy of a stable configuration with the C–C bond of a single ethoxy group perpendicular to the ring plane is approximately 600 cm^−1^ higher than that of a configuration with the C–C bond lying in the ring plane. Similarly, the stable configuration with both ethoxy C–C bonds perpendicular to the ring plane exhibits an electronic ground-state energy about 1200 cm^−1^ higher than that of the configuration with both bonds in the ring plane. Based on these results, it is predicted that the two lowest-energy rotamers, down–up and down–down, are likely to be observed experimentally.

The resonance-enhanced multiphoton ionization (REMPI) spectrum of m-diethoxybenzene was measured experimentally, and the hole-burning technique was used to resolve the spectra of different rotamers. Only two stable rotamers (down–up and down–down) were observed in the study. The REMPI spectra of each configuration were simulated theoretically, and good agreement was achieved with the measured results. With reference to the simulated spectra, the vibrational frequencies of the excited states of each configuration were assigned. Several in-plane vibrational modes of the benzene ring were observed, which were associated with the significant expansion of the ring during the transition from the ground state to the excited state. Other rotamers, characterized by higher energies and lower particle numbers, could not be observed in the supersonic molecular beam.

The MATI spectra of the down–up and down–down configurations were measured, and their accurate ionization energies were determined to be 62,419 cm^−1^ and 63,378 cm^−1^, respectively. The MATI spectra were also simulated theoretically, showing good consistency with the experimental results. With reference to the simulated spectra, the vibrational frequencies of the ionic ground states of each configuration obtained from the experiment were assigned. Several in-plane vibrational modes of the benzene ring were observed, which are related to the contraction of the ring during the transition from the excited state to the ionic ground state.

By comparing the results with m-dimethoxybenzene, the effect of different substituents on vibrational frequencies was discussed. The vibrational frequencies of m-diethoxybenzene are mostly higher than the corresponding frequencies of m-dimethoxybenzene, which may be attributed to the stronger electron-donating ability of the ethoxy group. This enhances the bonding interaction, resulting in a larger force constant. The vibrational frequencies of most ionic states are higher than the corresponding frequencies of the excited states; in particular, the vibrational frequency of the 18a mode in the cation is approximately 100 cm^−1^ higher than that in the corresponding excited state. This is associated with the contraction of the ring during the transition from the excited state to the ionic ground state, which shortens the equilibrium internuclear distance and increases the force constant. The corresponding vibrational frequencies of different rotational rotamers are usually very close; however, the orientation of substituents may also significantly affect the frequencies of a few vibrational modes. The frequency of the 6b mode in the down–down configuration of m-diethoxybenzene is approximately 88 cm^−1^ lower than that in the down–up configuration, and a similar trend is observed for the 6b mode in m-dimethoxybenzene.

## Figures and Tables

**Table 1:** The calculated relative zero-point-level energies of the ground-state rotamers (unit: cm^−1^).

Rotamer	B3LYP/6-311++g(d,p)	B3PW91/6-311++g(d,p)	B3LYP/aug-cc-pvtz	B3PW91/aug-cc-pvtz
Two ethoxy groups lying in the plane				
down–up–0–0	0	0	0	0
down–down–0–0	130	113	110	111
up–up–0–0	249	224	225	219
One ethoxy group being out of the plane				
down–up–out–0	592	548	599	586
down–up–0–out	594	550	604	592
down–down–out–0	712	670	698	686
up–up–out–0	856	806	845	829
Both ethoxy groups being out of the plane				
down–up–in–out	1182	1114	1203	1176
down–up–out–out	1186	1117	1206	1179
down–down–in–out	1296	1228	1287	1260
down–down–out–out	1296	1229	1288	1260
up–up–out–in	1468	1392	1471	1440
up–up–out–out	1487	1420	1482	1458

**Table 2:** Vibrational frequencies (in cm^−1^) and assignments of observed bands in the 2C-R2PI experiment of MDEB ^a^.

Down–Up			Down–Down			Assignment ^b^
Energy	Shift	Cal.	Energy	Shift	Cal.	
36,091	0	0	36,165	0	0	0^0^
36,196	105	102	36,282	117	108	βC_2_H_5_
36,250	159	158				βC_2_H_5_γC_2_H_5_^1^
36,261	170	165				10b^2^
36,282	191	182				βOC_2_H_5_
36,339	248					βOC_2_H_5_γOC_2_H_5_
36,391	300	293	36,463	298	290	9a^1^
36,477	386	384				15^1^
36,556	465	462				11^1^
36,625	534	532				6b^1^
36,640	549	553	36,693	528	530	6a^1^
36,820	729	731	36,895	730	730	1^1^
36,997	906	905	37,072	907	901	βCH (at CH_3_)
37,054	963	961	37,121	954	956	12^1^
37,092	1001	1017				18a^1^
37,245	1154					6a^1^9a^2^

^a^ The experimental values are shifts from 36,091 and 36,165 cm^−1^ for the down–up and down–down rotamers, respectively, whereas the predicted values are obtained from the B3PW91/aug-cc-pvtz calculations, scaled by 0.98 and 0.975, respectively. ^b^ β, in-plane bending; γ, out-of-plane bending.

**Table 3:** Vibrational frequencies (in cm^−1^) and assignments of observed bands in the MATI experiment of MDEB ^a^.

DOWN–UP				Down–Down		Assignment ^b^
Intermediate State				Intermediate State		
0	βC_2_H_5_	βOC_2_H_5_	Calc.	0	Calc.	
102	102		99	112	105	βC_2_H_5_
186	177	186	178			βOC_2_H_5_
299	287		298	298	295	9a^1^
				356	354	15^1^
405	403		407	399	397	16b^1^
520			528			6b^1^
591		593	592	579	582	6a^1^
				608	607	16b^1^10b^1^
741			740	736	735	1^1^
814				797	795	16b^2^
852	853		858			11^1^
				868		6a^1^9a^1^
895			898	895	896	νO-C_2_H_5_
974	997		972	969	967	12^1^
1014		1013		1009	1013	νO-CH_2_-CH_3_
1100	1113	1111	1102	1111	1111	18a^1^
1344			1344	1346	1349	13^1^
1531		1528	1536	1523	1534	8a^1^
1633			1638			1^1^νO-C_2_H_5_
1713			1716			11^2^

^a^ The experimental values are shifts from 62,419 and 63,378 cm^−1^ for down–down and down–up rotamers, respectively, whereas the predicted values are obtained from the B3PW91/aug-cc-pvtz calculations, scaled by 0.986 and 0.984, respectively. ^b^ ν, stretching; β, in-plane bending.

**Table 4:** The geometric parameters of the down–up rotamer of MDEB in the S_0_, S_1_, and D_0_ states calculated at B3PW91/aug-cc-pvtz, TD-B3PW91/aug-cc-pvtz, and UB3PW91/aug-cc-pvtz levels, respectively.

	S_0_	S_1_	D_0_	Δ(S_1_ − S_0_)	Δ(D_0_ − S_1_)
Bond length (Å)					
C1-C2	1.398	1.420	1.395	0.022	−0.025
C2-C3	1.387	1.415	1.382	0.028	−0.033
C3-C4	1.399	1.410	1.442	0.011	0.032
C4-C5	1.378	1.419	1.383	0.040	−0.036
C5-C6	1.394	1.416	1.379	0.022	−0.037
C6-C1	1.389	1.413	1.442	0.024	0.028
C1-O11	1.358	1.348	1.308	−0.010	−0.040
O11-C13	1.419	1.424	1.458	0.004	0.034
C13-C14	1.510	1.509	1.504	−0.001	−0.005
C3-O12	1.356	1.351	1.311	−0.006	−0.039
O12-C20	1.419	1.424	1.452	0.005	0.028
C20-C21	1.510	1.509	1.504	−0.001	−0.005
Bond angle (°)					
C1C2C3	119.81	115.94	118.68	−3.88	2.74
C2C3C4	120.13	122.34	120.56	2.20	−1.78
C3C4C5	119.18	120.99	120.30	1.81	−0.69
C4C5C6	121.73	117.46	119.81	−4.28	2.36
C5C6C1	118.58	120.64	119.86	2.06	−0.78
C6C1C2	120.56	122.46	120.79	1.90	−1.67
C6C1O11	124.48	123.28	122.70	−1.20	−0.58
C1O11C13	118.42	120.28	122.65	1.86	2.37
O11C13C14	107.96	107.94	107.49	−0.02	−0.45
C4C3O12	115.89	114.53	113.53	−1.36	−1.00
C3O12C20	118.60	119.53	120.50	0.93	0.97
O12C20C21	107.96	107.97	108.01	0.02	0.04
Dihedral angle (°)					
C2C1O11C13	−179.95	−178.60	−180	1.34	−1.40
C1O11C13C14	179.96	179.33	180	−0.64	0.67
C2C3O12C20	0	−1.89	0	−1.89	1.89
C3O12C20C21	179.99	−178.80	180	−1.30	1.20

**Table 5:** The geometric parameters of the down–down rotamer of MDEB in the S_0_, S_1_, and D_0_ states calculated at B3PW91/aug-cc-pvtz, TD-B3PW91/aug-cc-pvtz, and UB3PW91/aug-cc-pvtz levels, respectively.

	S_0_	S_1_	D_0_	Δ(S_1_ − S_0_)	Δ(D_0_ − S_1_)
Bond length (Å)					
C1-C2	1.389	1.415	1.386	0.026	−0.029
C2-C3	1.389	1.415	1.386	0.026	−0.029
C3-C4	1.396	1.415	1.446	0.019	0.031
C4-C5	1.387	1.417	1.381	0.030	−0.036
C5-C6	1.387	1.417	1.381	0.030	−0.036
C6-C1	1.396	1.415	1.446	0.019	0.031
C1-O11	1.357	1.348	1.309	−0.009	−0.039
O11-C13	1.419	1.423	1.455	0.004	0.032
C13-C14	1.510	1.509	1.504	−0.001	−0.005
C3-O12	1.357	1.348	1.309	−0.009	−0.039
O12-C20	1.419	1.423	1.455	0.004	0.032
C20-C21	1.510	1.509	1.504	−0.001	−0.005
Bond angle (°)					
C1C2C3	120.37	116.98	119.56	−3.39	2.58
C2C3C4	120.14	121.94	120.21	1.80	−1.73
C3C4C5	118.65	120.40	119.90	1.75	−0.50
C4C5C6	122.05	118.34	120.21	−3.71	1.87
C5C6C1	118.65	120.40	119.90	1.75	−0.50
C6C1C2	120.14	121.94	120.21	1.80	−1.73
C6C1O11	124.24	123.26	122.61	−0.98	−0.65
C1O11C13	118.59	120.31	122.71	1.72	2.40
O11C13C14	107.90	107.93	107.49	0.03	−0.44
C4C3O12	124.24	123.26	122.61	−0.98	−0.65
C3O12C20	118.59	120.31	122.71	1.72	2.40
O12C20C21	107.90	107.93	107.49	0.03	−0.44

**Table 6:** Fundamental frequencies of several vibrational modes for m-dimethoxybenzene and m-diethoxybenzene (unit: cm^−1^; theoretically calculated values in parentheses).

Molecule	Rotamer	State	6b	6a	1	12	18a
m-dimethoxybenzene *	down–up	S_1_	488 (463)	541 (550)	692 (693)	955 (938)	997 (1000)
		D_0_	485 (487)	572 (569)	715 (712)	961 (958)	1100 (1095)
	down–down	S_1_	(367)	(530)	693 (693)	954 (941)	(1033)
		D_0_	(402)	538 (545)	707 (709)	960 (957)	1103 (1107)
m-diethoxybenzene	down–up	S_1_	534 (532)	549 (553)	729 (731)	963 (961)	1001 (1017)
		D_0_	520 (528)	591 (592)	741 (740)	974 (972)	1100 (1102)
	down–down	S_1_	(444)	528 (530)	730 (730)	954 (956)	(1024)
		D_0_	(559)	579 (582)	736 (735)	969 (967)	1111 (1111)

* For the excited states, the calculated values are obtained at the CIS/6-311+G(d) level and scaled by 0.90. For the cationic ground states, the calculated values are obtained at the B3PW91/6-311++g(d,p) level and scaled by 0.98. For the measured values, see Ref. [43].

## Data Availability

The data that support the findings of this study are available from the corresponding author, Changyong Li, upon reasonable request.
